# Analysis of lens cloudiness during endoscopic submucosal dissection procedures: Effects of a novel lens cleaner

**DOI:** 10.1002/deo2.416

**Published:** 2024-07-28

**Authors:** Takashi Fujii, Sho Watanabe, Misugi Uga, Yuuki Matsui, Kazuomi Sakaki, Naoki Matsukawa, Tomoyo Machida, Masamichi Kurihara, Yoshihiro Tashiro, Eiko Okamoto, Tsunehito Yauchi, Shinji Suzuki, Shigeru Koyama

**Affiliations:** ^1^ Department of Gastroenterology Tokyo Metropolitan Hiroo Hospital Tokyo Japan; ^2^ Department of Gastroenterology Soka Municipal Hospital Saitama Japan; ^3^ Department of Endoscopy Tokyo Metropolitan Hiroo Hospital Tokyo Japan

**Keywords:** colorectal cancer, endoscope lens cloudiness, endoscopic submucosal dissection, gastric cancer, submucosal fat deposition

## Abstract

**Objectives:**

We aimed to identify independent factors for intraoperative endoscopic lens cloudiness during gastric and colorectal endoscopic submucosal dissections, investigate the effectiveness of Cleastay, an endoscope anti‐fog solution, and examine factors associated with severe submucosal fat deposition.

**Methods:**

A total of 220 patients who underwent gastric or colorectal endoscopic submucosal dissections in two institutions between January 2022 and October 2023 were included. Significant factors related to cloudiness were determined using univariate and multivariate analyses. Patient background and tumor characteristics related to severe submucosal fat deposition were investigated, and the degree of intraoperative endoscopic lens cloudiness and outcomes were compared between the Cleash and Cleastay groups.

**Results:**

In the multivariate analysis, factors increasing lens cloudiness included long procedure time (odds ratio [OR], 17.51; 95% confidence interval [CI], 1.52–202.08), stomach (vs. colon; OR, 5.08; 95% CI, 1.99–12.96), and severe submucosal fat deposition (OR, 12.19; 95% CI, 5.02–29.60). Conversely, the use of Cleastay (vs. Cleash; OR, 0.066; 95% CI, 0.021–0.21) was identified as a factor reducing cloudiness. Location analysis revealed that severe submucosal fat deposition was more common in the upper stomach and right colon.

**Conclusions:**

It was suggested that Cleastay is more useful for endoscopic submucosal dissection of the upper stomach and right colon, where severe submucosal fat deposition is expected.

## INTRODUCTION

Endoscopic submucosal dissection (ESD) has become a well‐established minimally invasive approach for superficial gastrointestinal neoplasms.^1^, ^2^, ^3^, ^4^, ^5^, ^6^, ^7^, ^8^ Although technically more difficult compared to conventional endoscopic mucosal resection (EMR), with potential adverse events such as delayed bleeding and perforation,^1^, ^2^, ^4^, ^8^ advancements in endoscopes and related instruments have improved its safety.^9^, ^10^, ^11^, ^12^, ^13^ Despite this, poor visibility due to endoscope lens cloudiness remains a common problem during ESD, leading to prolonged procedures and increased risks of adverse events.^14^, ^15^, ^16^, ^17^, ^18^ Studies have also reported an association between lens cloudiness during colorectal ESD and severe submucosal fat deposition,^14^, ^15^, ^18^ but similar studies for gastric ESD are lacking.

Lens cleaners are recommended to prevent lens cloudiness. SL cleaner (Sugiura Laboratory, Tokyo, Japan, Cleash), containing a surfactant and ethanol, is Japan's most widely used anti‐fog solution. Yoshida et al. demonstrated the superior efficacy of Cleash (Nagase Medicals Co., Ltd.), containing two types of non‐ionic surfactants and pharmaceutical excipients, compared to SL cleaner during colorectal ESD.^14^ Cleastay (Neuroceuticals) was recently introduced as an anti‐fog solution containing a 2‐methacryloyloxyethyl phosphorylcholine (MPC) polymer; however, its effectiveness compared to existing anti‐fog solutions remains unclear.

Therefore, this study aimed to identify independent factors related to endoscope lens cloudiness during gastric and colorectal ESD, investigate the effectiveness of Cleastay compared to other solutions, and evaluate the factors that contribute to severe submucosal fat deposition.

## METHODS

### Patients

This was a retrospective, multicenter study including 220 patients who underwent gastric or colorectal ESD at either Tokyo Metropolitan Hiroo Hospital (*n* = 118) or Soka City Hospital (*n* = 102) between January 2022 and October 2023. The indications for ESD followed the guidelines of the Japan Gastroenterological Endoscopy Society.^19^, ^20^

### ESD procedure

ESD procedures in this study were performed by an expert or a trainee under expert supervision. The procedure was performed under intravenous sedation with midazolam and pethidine hydrochloride. The GIF‐H290T (Olympus Medical Systems Co.) endoscope was used for gastric ESDs, whereas the PCF‐H290T (Olympus Medical Systems Co.) endoscope was used for colorectal ESDs. High‐frequency generation was provided by the VIO300D (Erbe Elektromedizin). Endocut I mode was used for mucosal incision and swift coagulation mode was used for submucosal dissection. Soft coagulation mode was used for intraoperative bleeding and vascular treatment of post‐resection ulcers. Gastric ESD used the Elastic Touch slit‐&‐hole type tip hood (F040; TOP Corporation), while colorectal ESD used the short ST hood (Fujifilm Medical Co., Ltd.). Furthermore, Cleash and Cleastay, the lens cleaners, were used prior to the endoscopic insert according to the instructions in the package insert. Cleash was used from January 2022 to February 2023, and Cleastay was used for all patients after February 2023, when Cleastay became available.

The ESD procedure was performed as follows. A Flush Knife BT (Fujifilm Medical Co.) was used to mark approximately 5 mm beyond the tumor margin. Next, 0.4% hyaluronic acid solution (Ksmart; Olympus Medical Systems Co.) with minimal indigo carmine was injected into the submucosal tissue using a 25G endoscopic puncture needle (TOP Corporation). Subsequently, mucosal incision and submucosal dissection were performed from the proximal side of the tumor for gastric ESD and from the distal side for colorectal ESD.

### Data analysis

The medical records of the 220 patients were reviewed to extract the following data: age, sex, comorbidities (hypertension, hyperlipidemia, and diabetes mellitus), lesion site, lesion shape, lesion size, type of anti‐fog solution, procedure time, complications (perforation and delayed bleeding), en bloc resection rate, histologic complete resection rate, and operator (expert or trainee).

Lesion sites and shapes were classified according to the Japanese Classification of Gastric Carcinoma and the Japanese Classification of Colorectal, Appendiceal, and Anal Carcinoma.^21^, ^22^ Operators were classified as experts if they had performed ≥200 gastric ESDs and ≥80 colorectal ESDs or as trainees if they had performed <200 gastric ESDs or <80 colorectal ESDs.^23^, ^24^

Intraoperative procedures were recorded as anonymized endoscopic videos (no personal information). Three experts watched all the video recordings and graded the level of endoscope cloudiness and the amount of submucosal fat based on the highest cloudiness level and the greatest amount of submucosal fat.

**FIGURE 1:**
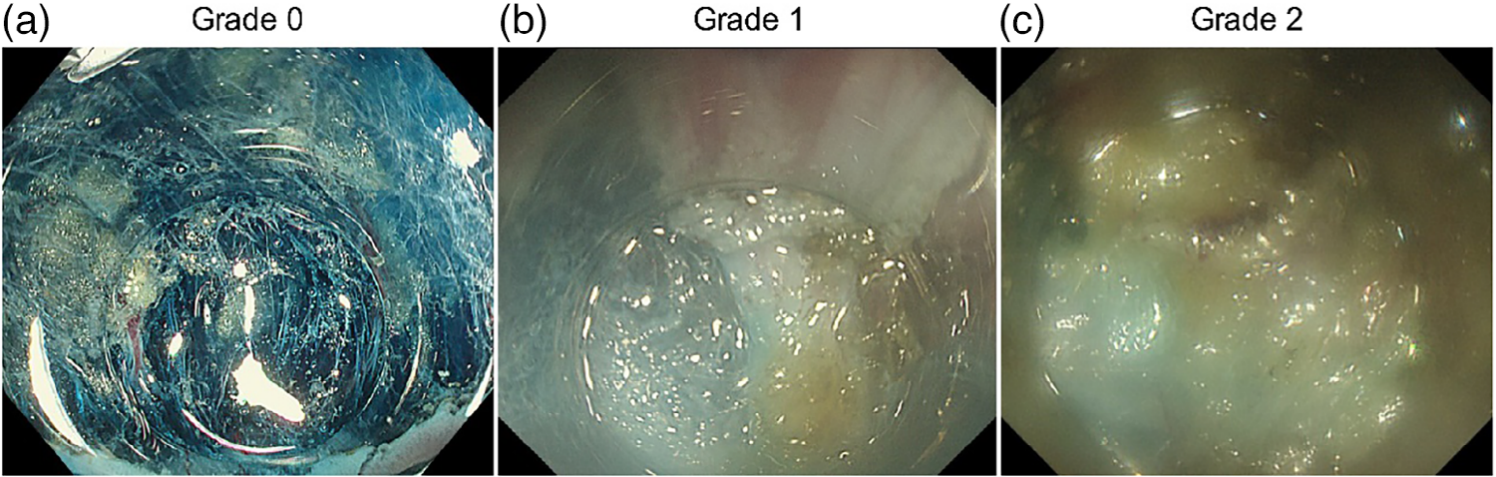
Visibility scores. The degree of endoscope lens cloudiness was scored as follows: (a) Grade 0 was defined as a condition in which the endoscope lens was fog‐free or, if it was cloudy, it could be easily cleaned by spraying water; (b) Grade 1 was defined as a condition in which the endoscope lens could not be defogged by spraying water, but the cloudiness did not prevent mucosa observation; and (c) Grade 2 was defined as a condition in which the endoscope lens was so cloudy that it could not be defogged by spraying water and the mucosa could not be observed.

Following previous studies, endoscope lens cloudiness level was scored from Grades 0–2.^14^, ^15^ Grade 0 indicated no cloudiness or easily removable cloudiness with water spray (Figure 1a), Grade 1 indicated cloudiness that could not be removed but allowed observation (Figure 1b), and Grade 2 indicated severe cloudiness that could not be removed and completely prevented observation (Figure 1c). For intraoperative cloudiness, water spray was used to remove the cloudiness, but if the cloudiness was Grade 2, observation was difficult; thus, the endoscope was removed, the lens was wiped, and lens cleaner was applied again.

**FIGURE 2:**
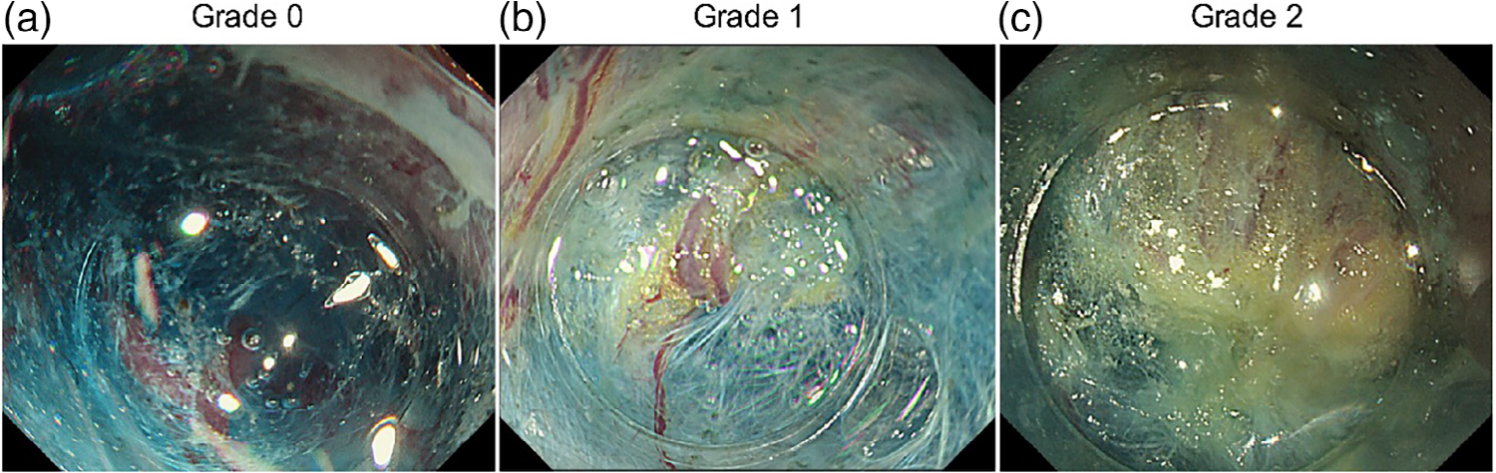
Degree of deposition of submucosal fatty tissue. (a) Grade 0, no fatty tissue in the submucosal layer; (b) Grade 1, small cotton or dot‐like faint fatty tissue in the submucosal layer; and (c) Grade 2, the submucosal layer is almost covered with thick fatty tissue.

Submucosal fat was also scored from Grades 0–2, following previous studies.^14^, ^15^ Grade 0 indicated the absence of fat (Figure 2a); Grade 1 indicated mild to moderate fat deposition, evident as streaks or spots of fat (Figure 2); and Grade 2 indicated severe fat deposition, evident as thick sheets of fat (Figure 2c).

### Study endpoints

The primary outcome was the analysis of factors contributing to endoscope lens cloudiness during gastric and colorectal ESD. The secondary outcome was the comparison and analysis of the clinical effectiveness of Cleastay versus Cleash.

### Statistical analysis

Data are presented as absolute numbers (%) and means ± standard deviations. Categorical variables were compared using the chi‐square test or Fisher's exact test, whereas continuous variables were compared using Wilcoxon's rank sum test. Multiple logistic regression analysis was performed to evaluate factors affecting lens cloudiness. All statistical analyses were performed using JMP version 17.0.0 (SAS Institute, Inc.), and two‐tailed statistical significance was set at *p* < 0.05.

## RESULTS

### Patient characteristics

The study included 96 (43.6%) and 124 (56.4%) patients who underwent gastric and colorectal ESDs, respectively, with a median age of 76.0 years. Cleastay was used in 71 patients (32.3%), whereas Cleash was used in 149 (67.7%). Lens cloudiness was Grade 0 in 160 patients (72.7%) and Grade 1/2 in 60 (27.3%). En‐bloc resection was achieved in 218 patients (99.1%), with positive or unknown resection margins in 10 (4.5%) and complications in five (2.3%; Table 1).

**TABLE 1:** Univariate analysis of the relation between patients’ background, clinical characteristics, clinical outcomes, and lens cloudiness.

	Total (*n* = 220)	Grade 1/2 (*n* = 60)	Grade 0 (*n* = 160)	*p*‐value
**Characteristics**				
Age (years.), median [IQR]	76.0 [71.0, 82.0]	76.0 [72.0, 82.0]	76.0 [70.3, 82.0]	0.4937
Sex (male), *n* (%)	137 (62.3%)	41 (68.3%)	96 (60.0%)	0.2523
BMI, median [IQR]	23.1 [20.7, 25.6]	23.8 [20.8, 25.2]	23.0 [20.7, 26.0]	0.8379
Diabetes mellitus, *n* (%)	50 (22.7%)	14 (23.3%)	36 (22.5%)	0.8957
Hypertension, *n* (%)	149 (67.7%)	40 (66.7%)	109 (68.1%)	0.8370
Hyperlipidemia, *n* (%)	98 (44.5%)	32 (53.3%)	66 (41.3%)	0.1090
Organ, *n* (%)				
Stomach	96 (43.6%)	42 (70.0%)	54 (33.7%)	<0.0001^*^
Colon	124 (56.4%)	18 (30.0%)	106 (66.3%)	
Location (stomach), *n* (%)				
Upper part	23 (24.0%)	15 (35.7%)	8 (14.8%)	0.0017^*^
Middle part	34 (35.4%)	18 (42.9%)	16 (29.6%)	
Lower part	39 (40.6%)	9 (21.4%)	30 (55.6%)	
Location (colon), *n* (%)				
Right‐sided	72 (58.1%)	11 (61.1%)	61 (57.5%)	0.7763
Left‐sided	52 (41.9%)	7 (38.9%)	45 (42.5%)	
Tumor size (mm), median [IQR]	22.0 [18.3, 29.0]	22.5 [15.0, 31.8]	22.0 [20.0, 28.0]	0.8890
Submucosal fatty tissue, *n* (%)				
Grade 0/1	141 (64.1%)	16 (26.7%)	125 (78.1%)	<0.0001^*^
Grade 2	79 (35.9%)	44 (73.3%)	35 (21.9%)	
Lens cleaner, *n* (%)				
Cleastay	71 (32.3%)	6 (10.0%)	65 (40.6%)	<0.0001^*^
Cleash	149 (67.7%)	54 (90.0%)	95 (59.4%)	
Endoscopist, expert, *n* (%)	87 (39.5%)	14 (23.3%)	73 (45.6%)	0.0020^*^
**Outcomes**				
Procedure time (min), median [IQR]	60.0 [40.0, 94.8]	86.0 [58.0, 103.8]	51.0 [36.3, 80.0]	<0.0001^*^
En bloc resection, *n* (%)	218 (99.1%)	58 (96.7%)	160 (100%)	0.0735
Histologic complete resection, *n* (%)	210 (95.5%)	51 (85.0%)	159 (99.4%)	<0.0001^*^
Adverse events, *n* (%)	5 (2.3%)	5 (8.3%)	0 (0%)	0.0013^*^

Abbreviations: BMI, body mass index; IQR, interquartile range.

Adverse events: perforation in three patients and delayed bleeding in two patients in the Cleash group.

※*p* < 0.05.

### Investigation of factors affecting lens cloudiness

Table 1 shows the results of the univariate analysis of lens cloudiness and patient characteristics/clinical results. No significant differences were observed in the body mass index (BMI; 23.8 kg/m^2^ vs. 23.0 kg/m^2^, *p* = 0.8379) and diabetes mellitus history (23.3% vs. 22.5%, *p* = 0.8957) between patients with Grades 1 or 2 and 0 cloudiness. The proportion of gastric ESD procedures in which Grade 1/2 cloudiness occurred was significantly higher than that of colorectal ESD (70.0% vs. 33.7%, *p* < 0.0001), with significant differences in lesion distribution within the stomach (upper, middle, or lower; *p* = 0.0017). Furthermore, more patients with severe submucosal fat deposition versus no submucosal fat deposition had Grade 1/2 cloudiness (73.3% vs. 21.9%, *p* < 0.0001). The latter group also used Cleash more often (90.0% vs. 59.4%, *p* < 0.0001), and the operator was more likely a trainee (76.7% vs. 54.4%, *p* = 0.0020).

Clinically, compared to those with Grade 0 cloudiness, patients with Grade 1/2 cloudiness demonstrated longer mean procedure times (86.0 min vs. 51.0 min, *p* <0.0001), higher proportions of achieving positive or unknown resection margins (15.0% vs. 0.6%, *p* <0.0001), and higher complication incidences (8.3% vs. 0%, *p* = 0.0013).

### Assessment of factors affecting lens cloudiness

Multiple logistic regression analysis was performed (Grade 1/2 or Grade 0) with the significant factors in the univariate analysis; lens cloudiness was the dependent variable, and procedure time, organ, submucosal fat deposition (Grade 0/1 or Grade 2), anti‐fog solution type, and operator expertise were independent variables (Table 2). The results showed that longer procedure times significantly increased lens cloudiness (odds ratio [OR], 17.51; 95% confidence interval [CI], 1.52–202.08). The organ is the stomach also increased lens cloudiness (OR, 5.08; 95% CI, 1.99–12.96), and submucosal fat deposition (Grade 2) also significantly increased lens cloudiness (OR, 12.19; 95% CI, 5.02–29.60), whereas Cleastay use significantly reduced it (OR, 0.066; 95% CI, 0.021–0.21). Conversely, no significant differences were observed in operator expertise.

**TABLE 2:** Multivariate analysis of the clinical factors related to lens cloudiness (95％ confidence interval [CI]).

	OR [95% CI]	*p‐*value
Procedure time (range)	17.51 [1.52, 202.08]	0.0218^*^
Stomach (vs. colon)	5.08 [1.99, 12.96]	0.0007^*^
Submucosal fatty tissue Grade 2 (vs Grade 0/1)	12.19 [5.02, 29.60]	<0.0001^*^
Cleastay (vs. leash)	0.066 [0.021, 0.21]	<0.0001^*^
Expert (vs. trainee)	1.35 [0.49, 3.70]	0.5587

※*p* < 0.05.

### Investigation of the clinical effectiveness of different anti‐fog solutions

Table 3 shows a comparison of different anti‐fog solutions in gastric ESDs. The Cleastay group had less cloudiness (20.8% vs. 51.4%, *p* = 0.0098) than the Cleash group, but no significant differences were observed in the clinical results (procedure time, *en bloc* resection rate, histologic complete resection rate, and complications).

**TABLE 3:** **Stomach**: Comparison of the patients’ background, clinical characteristics, and clinical outcomes between the Cleastay and Cleash groups.

	Cleastay (*n* = 24)	Cleash (*n* = 72)	*p‐*value
**Characteristics**			
Location, *n* (%)			
Upper part	9 (37.5%)	14 (19.4%)	0.2203
Middle part	7 (29.2%)	27 (37.5%)	
Lower part	8 (33.3%)	31 (43.1%)	
Tumor size (mm), median [IQR]	20.0 [10.5, 33.8]	18.0 [10.0, 28.0]	0.2644
Submucosal fatty tissue, *n* (%)			
Grade 0/1	11 (45.8%)	40 (55.6%)	0.4088
Grade 2	13 (54.2%)	32 (44.4%)	
Endoscopist, expert, *n* (%)	7 (29.2%)	10 (13.9%)	0.1032
**Outcomes**			
Lens cloudiness, *n* (%)			
Grade 0	19 (79.2%)	35 (48.6%)	0.0098^*^
Grade 1/2	5 (20.8%)	37 (51.4%)	
Procedure time (min), median [IQR]	67.5 [46.3, 103.0]	58.0 [35.3, 89.3]	0.1587
En bloc resection, *n* (%)	24 (100%)	71 (98.6%)	1.0000
Histologic complete resection, *n* (%)	24 (100%)	66 (91.7%)	0.3315
Adverse events, *n* (%)	0 (0%)	4 (5.6%)	0.5691

Abbreviation: IQR, interquartile range.

Adverse events: perforation in two patients and delayed bleeding in two patients in the Cleash group.

※*p* < 0.05.

Table 4 shows a comparison of different anti‐fog solutions in colorectal ESD. The Cleastay group demonstrated a higher likelihood of experts as the operator (74.5% vs. 45.5%, *p* = 0.0013), less cloudiness (2.1% vs 22.1%, *p* = 0.0015), shorter procedure times (54.0 min vs. 65.0 min, *p* = 0.0456) than the Cleash group.

**TABLE 4:** **Colon**: Comparison of the patients’ background, clinical characteristics, and clinical outcomes between the Cleastay and Cleash groups.

	Cleastay (*n* = 47)	Cleash (*n* = 77)	*p‐*value
**Characteristics**			
Location, *n* (%)			
Right‐sided	25 (53.2%)	47 (61.0%)	0.3909
Left‐sided	22 (46.8%)	30 (39.0%)	
Tumor size (mm), median [IQR]	23.0 [20.0, 28.0]	24.0 [21.0, 30.5]	0.2615
Submucosal fatty tissue, *n* (%)			
Grade 0/1	31 (66.0%)	59 (76.6%)	0.1997
Grade 2	16 (34.0%)	18 (23.4%)	
Endoscopist, expert, *n* (%)	35 (74.5%)	35 (45.5%)	0.0013^*^
**Outcomes**			
Lens cloudiness, *n* (%)			
Grade 0	46 (97.9%)	60 (77.9%)	0.0015^*^
Grade 1/2	1 (2.1%)	17 (22.1%)	
Procedure time (min), median [IQR]	54.0 [40.0, 69.0]	65.0 [40.0, 117.0]	0.0456^*^
En bloc resection, *n* (%)	49 (100%)	76 (98.7%)	1.0000
Histologic complete resection, *n* (%)	47 (100%)	73 (94.8%)	0.2964
Adverse events, *n* (%)	0 (0%)	1 (1.3%)	1.0000

Abbreviation: IQR, interquartile range.

Adverse event: perforation in one patient in the Cleash group.

※*p* < 0.05.

### Assessment of factors affecting severe submucosal fat deposition

Table 5 shows a comparison of patient characteristics and tumor locations between those with and without severe submucosal fat deposition in the stomach. There was more severe submucosal fat deposition in the upper stomach compared to that in the lower stomach (upper vs. lower stomach, *p* < 0.0001).

**TABLE 5:** **Stomach**: Comparison between groups by presence or absence of submucosal fat deposition.

	Grade 2 (*n* = 45)	Grade 0/1 (*n* = 51)	*p‐*value
Age (years), median [IQR]	77.0 [73.5, 83.0]	79.0 [75.0, 85.0]	0.1402
Sex (male), *n* (%)	31 (68.9%)	32 (62.8%)	0.5265
BMI, median [IQR]	24.6 [21.6, 26.6]	23.0 [19.8, 25.4]	0.1008
Diabetes mellitus, *n* (%)	11 (24.4%)	12 (23.5%)	0.9165
Hypertension, *n* (%)	27 (60.0%)	39 (76.5%)	0.0819
Hyperlipidemia, *n* (%)	21 (46.7%)	28 (54.9%)	0.4203
Location (stomach), *n* (%)			
Upper part	16 (35.5%)	7 (13.7%)	<0.0001^*^
Middle part	21 (46.7%)	13 (25.5%)	
Lower part	8 (17.8%)	31 (60.8%)	

Abbreviations: BMI, body mass index; IQR, interquartile range

※*p* < 0.05.

Table 6 shows a comparison of patient characteristics and tumor locations between those with and without severe submucosal fat deposition in the colon. The right colon had more severe submucosal fat deposition than the left colon (right vs. left colon, *p* = 0.0005).

**TABLE 6:** **Colon**: Comparison between groups by presence or absence of submucosal fat deposition.

	Grade 2 (*n* = 34)	Grade 0/1 (*n* = 90)	*p‐*value
Age (years), median [IQR]	75.5 [65.3, 77.3]	74.5 [70.0, 81.0]	0.0606
Sex (male), *n* (%)	20 (58.8%)	54 (60.0%)	0.9052
BMI, median [IQR]	24.2 [20.1, 25.7]	22.4 [20.7, 25.0]	0.3438
Diabetes mellitus, *n* (%)	8 (23.5%)	19 (21.1%)	0.7723
Hypertension, *n* (%)	26 (76.5%)	57 (63.3%)	0.1572
Hyperlipidemia, *n* (%)	13 (38.2%)	36 (40.0%)	0.8575
Location (colon), *n* (%)			
Right‐sided	28 (82.4%)	44 (48.9%)	0.0005^*^
Left‐sided	6 (17.6%)	46 (51.1%)	

Abbreviations: BMI, body mass index; IQR, interquartile range

※*p* < 0.05.

## DISCUSSION

Endoscope lens cloudiness is a common issue that hinders visualization during gastric and colorectal ESDs, resulting in operator stress and increased procedural difficulty.^14^, ^15^, ^16^, ^17^, ^18^ While studies have reported its association with severe submucosal fat deposition in colorectal ESD,^14^, ^18^ to our knowledge, no study has investigated the factors contributing to cloudiness during gastric ESD.

In this study, we conducted multivariate analyses of factors associated with endoscope lens cloudiness during gastric and colorectal ESDs, Cleastay significantly reduced cloudiness, and the long procedure time, stomach (vs. colon), and severe submucosal fat deposition increased cloudiness.

Our findings are inconsistent with those of Yoshida et al.,^14^ who suggested that Cleash was more effective than SL cleaner. Cleastay is a novel anti‐fog solution that does not affect the human body and ensures good visibility by utilizing an MPC polymer film coat, a different mechanism from that of surfactant‐based lens cleaners. The film's high biocompatibility, due to its similar molecular structure with phospholipids, minimizes protein and blood cell adhesion.^25^ In addition to its resistance to protein and blood cell adhesion, the structure of Cleastay enables “self‐cleaning” due to the hydrophilic effect of being washed away with water and anti‐cloudiness due to that same effect with water vapor.^25^ However, despite its perceived utility in endoscopy, this has not been reported in existing literature. In the present study, Cleastay use significantly reduced endoscope lens cloudiness during gastric and colorectal ESDs, which may be attributed to the resistance of the MPC polymer to protein absorption and its self‐cleaning function, as previously discussed. The clinical value of Cleastay was further investigated by stratifying the Cleastay and Cleash groups according to the organ. In gastric ESD, the Cleastay group showed significantly less cloudiness. Similarly, in colorectal ESD, the Cleastay group showed significantly less cloudiness, shorter procedure times, and a higher proportion of achieving negative resection margins. However, there is a potential bias due to the higher proportion of experts in this group. Unfortunately, we did not conduct a subgroup analysis to compare the results of experts and trainees in both groups, and the effect of Cleastay on clinical treatment was difficult to assess. Large‐scale prospective studies are required to clarify and further investigate the impact of different lens cleaners on clinical results.

Furthermore, long procedure time was an associated factor for lens cloudiness. Long procedure time is reportedly increased by difficult hemostasis procedures and poor site‐specific maneuverability.^26^, ^27^, ^28^, ^29^ Long procedure times are thought to cause lens cloudiness because hemostatic procedures and unstable vision tend to cause protein and oil adherence.

Multivariate analysis revealed more severe lens cloudiness in the stomach than in the large intestine. In addition, the location‐specific analysis of the stomach revealed more severe lens cloudiness in the upper stomach compared to that in the lower stomach. There have been no reports comparing lens cloudiness during gastric and colorectal ESD procedures or comparing lens cloudiness in different locations in the stomach. Intraoperative bleeding is a common complication of gastric ESD, especially in the upper stomach, which is reportedly a risk factor for intraoperative bleeding due to the presence of larger blood vessels and an unstable visual field compared to other locations ^26^, ^27^, ^28^, ^30^, ^31^ Furthermore, as discussed below and shown in Table 5, severe submucosal fat deposition is abundant in the upper stomach, and severe submucosal fat deposition has also been demonstrated in the multivariate analysis of this study as a factor in lens cloudiness. Based on the above, we believe that the stomach, especially the ESD in the upper stomach, where an unstable field of view with hemorrhage develops, is easily clouded by protein and oil adhesion.

Additionally, severe submucosal fat deposition was found to significantly increase lens cloudiness, which was consistent with previous studies on colorectal ESD.^14^, ^18^ During dissection and coagulation in areas with submucosal fat deposition, oily components may adhere to the lens, and inadequate thermal coagulation can exacerbate coagulation,^17^ all of which contribute to the endoscope lens cloudiness from fat deposition. Yoshida et al.^14^ reported that BMI was associated with submucosal fat deposition, while Tanaka et al.^15^ demonstrated that BMI ≥ 25 kg/m^2^, HbA1c ≥ 6.5%, and right colon localization were predictive factors for severe submucosal fat deposition. In the present study, differences in submucosal fat deposition were observed based on the tumor location. Severe submucosal fat deposition was more common in the upper part of the stomach, and the right side of the colon. On the other hand, BMI and diabetes mellitus were not associated with severe submucosal fat deposition. We believe that the results varied from those of previous reports as there were more cases of severe submucosal fat deposition in the present study compared to those in the report by Yoshida et al.^14^ (8.7% of severe submucosal fat deposition in the report by Yoshida et al.^14^ and 35.9% in the present study) and that the classification was not made in the form of BMI ≥ 25 kg/m^2^ and HbA1c ≥ 6.5%, unlike that in the report by Tanaka et al.^15^ Thus, the presence of severe submucosal fat deposition may be more related to tumor location than to patient background; however, since no studies have examined the differences in submucosal fat deposition based on the location of the stomach, future results should be confirmed in large prospective studies with matched background factors. Although consistent with previous reports, our finding that severe submucosal fat deposition was more common in the right colon than in the left did not translate to a significant difference in lens cloudiness. This may be due to insufficient power in the study, given the low incidence of Grade 1/2 endoscope lens cloudiness during colorectal ESD (18/106, 16.9%). Studies with larger sample sizes are required to confirm this finding.

Despite the insights offered by this study, several limitations should be acknowledged. First, the non‐blinded retrospective nature of the study introduces potential bias with variations in patient characteristics. This is further exacerbated by variations in performing ESD between institutions, although we attempted to mitigate this bias by combining the clinical results of the two institutions. Further large‐scale, multicenter studies are required to confirm our results. Second, the evaluations of lens cloudiness and the amount of submucosal fat deposition were subjective, as they were based on assessments made by multiple experts who viewed the anonymized videos. The development of objective and quantitative indices for lens cloudiness and the amount of fat deposition would improve the assessment of these parameters. Additionally, large‐scale, randomized, double‐blind, prospective studies would strengthen this research. Finally, most colorectal ESD cases in the Cleastay group were managed by experts, resulting in potential expert bias. Future analyses should compare experts and trainees to accurately assess the treatment impact.

In conclusion, it was suggested that Cleastay is more useful for ESD of the upper stomach and right colon, where severe submucosal fat deposition is expected.

## CONFLICT OF INTEREST STATEMENT

None.

## ETHICS STATEMENT

This study was approved by the Tokyo Metropolitan Hiroo Hospital Institutional Review Board (Expedited‐21) and was conducted in accordance with the Declaration of Helsinki. All patients were informed about the risks and benefits of ESD and provided written informed consent before enrollment.
